# Grinding Wheel Loading Evaluation by Using Acoustic Emission Signals and Digital Image Processing

**DOI:** 10.3390/s20154092

**Published:** 2020-07-22

**Authors:** Chien-Sheng Liu, Yang-Jiun Ou

**Affiliations:** 1 Department of Mechanical Engineering, National Cheng Kung University, Tainan City 70101, Taiwan; 2 Department of Mechanical Engineering, National Chung Cheng University, Chiayi County 62102, Taiwan; v5124v521@gmail.com

**Keywords:** wheel loading, wheel wear, grinding, acoustic emission, continuous generating gear grinding machine, image processing

## Abstract

In the manufacturing industry, grinding is used as a major process for machining difficult-to-cut materials. Grinding is the most complicated and precise machining process. For grinding machines, continuous generating gear grinding machines are widely used to machine gears which are essential machine elements. However, due to its complicated process, it is very difficult to design a reliable measurement method to identify the grinding wheel loading phenomena during the grinding process. Therefore, this paper proposes a measurement method to identify the grinding wheel loading phenomenon in the grinding process for continuous generating gear grinding machines. In the proposed approach, an acoustic emission (AE) sensor was embedded to monitor the grinding wheel conditions; an offline digital image processing technique was used to determine the loading areas over the surface of Al_2_O_3_ grinding wheels; and surface roughness of the ground workpiece was measured to quantify its machining quality. Then these three data were analyzed to find their correlation. The experimental results have shown that there are two stages of grinding in the grinding process and the proposed measurement method can provide a quantitative grinding wheel loading evaluation from the AE signals online.

## 1. Introduction

In machining operations, for cutting hardworking materials and precise gears, grinding process is a standard and dominative process due to its intrinsic and efficient machining mechanism [1]. Grinding process is also used as the final machining step to produce a precise workpiece with good surface finish and accurate sizes [2]. It means that the failure of the grinding process may lead to irreparable result for the ground workpiece and increase extra manufacturing cost [3]. In order to ensure the grinding efficiency and avoid mechanical defects in the grinding process, the surface condition and surface morphology of the grinding wheel are most critical factors [4,5]. In other words, tool wear (or grinding wheel loading) monitoring is crucial during grinding process to save cost and improve efficiency [6].

For grinding process, grinding wheel loading is too common to maintain good the surface condition and surface morphology of the grinding wheel. Therefore, grinding wheel loading might decrease the surface finish of the ground workpiece and the grinding efficiency [7]. In the grinding process, this phenomenon is known as “grinding wheel loading”, which can be defined as the state of a grinding wheel when debris of a workpiece either adheres to the abrasive grains, is weld to the top of abrasive grains or becomes embedded in the spaces between abrasive grains on grinding wheels [8,9]. This phenomenon leads to excessive vibration and rubbing because dull wheel grains deteriorate wheel cutting ability [9,10,11]. It also increases the grinding force, temperature, and grinding burn and reduces the grinding wheel life [9,10,11,12,13]. Therefore, monitoring the grinding wheel loading phenomena and developing a reliable online monitoring technique during the grinding process play a key role in the quality of workpieces being manufactured. Acoustic emission (AE) is an efficient method that monitors the transient vibrational waves generated by the rapid release of energy from localized sources, e.g., fracture, within a material. It has been thought as a most efficient method to monitor the grinding process online due to its superior sensitivity [14,15,16]. The AE generated during machining and grinding processes has been proved to be related to the process state and to the surface condition of the tool and workpiece [17,18]. Therefore, AE technology has been widely used as a non-destructive inspection method for monitoring a wide variety of machining processes and industrial applications [19], including grinding [20,21,22,23,24,25,26,27,28,29,30], turning [31,32,33], electro-discharge grinding [20], sawing [34], abrasive water jet machining [35], rock bridge stability [36], welding [37], vibroarthrography [38], monitoring of concrete materials [39,40], leakage [41], and power system [42].

In the literature, various monitoring methods have been used for the online evaluation of grinding processes for traditional flat surface-grinding machine tools [7,8,9,10,11,12,13,14,15,16,17,18]. In a previous paper [13], the present group proposed a measurement method based on the AE technique to characterize the loading phenomena of a Si_2_O_3_ grinding wheel for a traditional flat surface-grinding machine tool. However, with the constantly expanding market of gears, continuous generating gear grinding machines gain rising importance [43]. It is necessary to develop a monitoring method for the online evaluation of grinding processes for continuous generating gear grinding machines. Consequently, in the present paper, a measurement method is further developed to identify the grinding wheel loading phenomenon in the grinding process for continuous generating gear grinding machines. The performance of the proposed method is verified experimentally using a commercial continuous generating gear grinding machine. In the proposed approach, an AE sensor was embedded to monitor the grinding wheel conditions. In this manner, the AE_RMS_ (AE root-mean-square) signal of the AE sensor was collected through the grinding process of the continuous generating gear grinding machine. At the same time, an offline digital image processing technique was used to determine the loading areas over the surface of Al_2_O_3_ grinding wheels; and surface roughness of the ground workpiece was measured to quantify its machining quality. Then these three data were used to find their correlation by applying a data analysis method. The experimental results show that the proposed measurement method can provide an early warning quantitative index for grinding wheel loading evaluation and monitoring from the AE signals online.

## 2. Proposed Measurement Methodology and Experimental Setup

**Figure 1:**
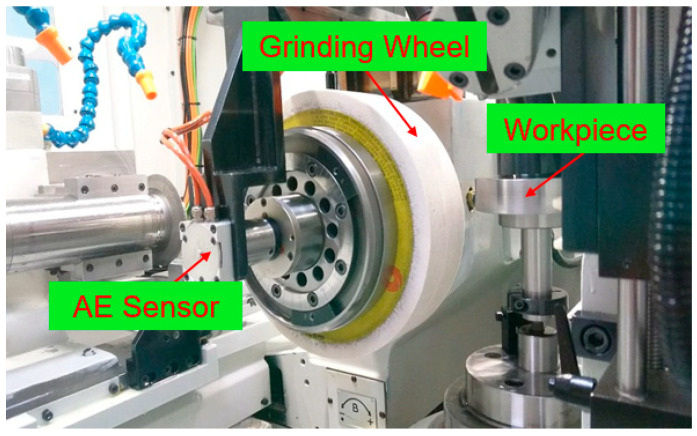
Photograph of continuous generating gear grinding machine and workpiece.

**Figure 2:**
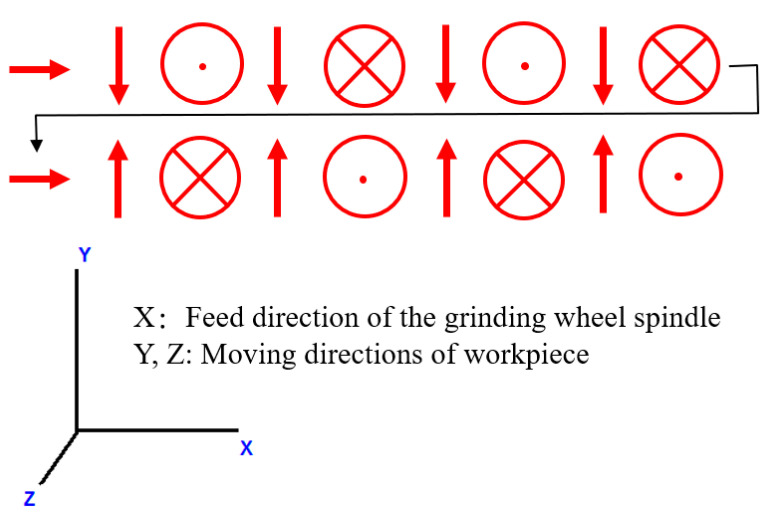
Schematic diagram of grinding path.

Figure 1 shows the photograph of the experimental setup. In this paper, the experiments are performed on a LGA-2812 (Luren, Hsinchu City, Taiwan) continuous generating gear grinding machine with an Al_2_O_3_ grinding wheel (Model: 1A32A120J8V, Kinik, New Taipei City, Taiwan). An AE sensor (Model: VM25, Balance Systems, MI, Italy, sampling rate of 20 Hz) is embedded into the spindle of the grinding wheel to pick up the AE signals. In this paper, a specific grinding path, as shown in Figure 2, was designed to accelerate and investigate the grinding wheel loading phenomena with cylindrical grinding wheel and workpiece. The symbols “→” and “↑” indicate the feed direction of the grinding wheel spindle along x axial and the moving direction of the workpiece along y axial, respectively. The symbols “⊙” and “⊕” indicate the moving directions of the workpiece along + z axial and − z axial, respectively. This grinding path considers the continuous generating gear grinding and well-distributed grinding wheel loading. The experimental parameters are decided according to the relevant experiences of workers and the variable parameters of the machine. Table 1 lists these experimental parameters. It is noted that the workpiece axis speed is automatically adjusted according to the grinding wheel speed by using the machine’s software.

**Figure 3:**
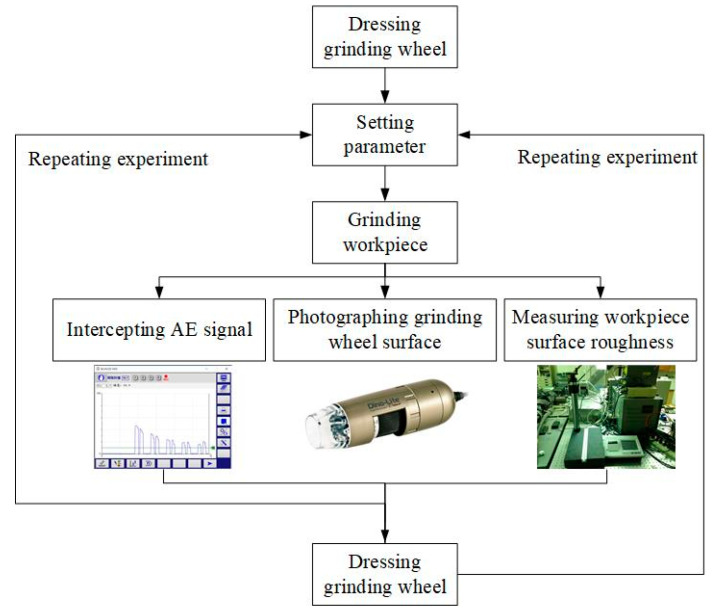
Flow chart of grinding procedure.

The flowchart of the grinding procedure is shown in Figure 3. In the proposed measurement method, an AE sensor was embedded to monitor the grinding wheel conditions. Here, the AE_RMS_ signal of the AE sensor was collected through the grinding process of the continuous generating gear grinding machine. Equation (1) formulates the AE_RMS_ signal:

(1) $${\mathrm{AE}}_{\mathrm{RMS}}=\sqrt{\frac{1}{\Delta T}\int_{0}^{\Delta T}{{\mathrm{AE}}_{V}}^{2}(t)dt}$$

where Δ*T* is the integration time constant, and AE_V_ is the instantaneous signal [5,12]. In the present paper, the AE_RMS_ signal of the AE sensor was used to average the random signals and then to induce the grinding performance easily. It is noted that Wang et al. (2001) and Liu et al. (2018) provide a more comprehensive description of the AE_RMS_ signal for traditional flat surface-grinding machine tools [13,17].

**Figure 4:**
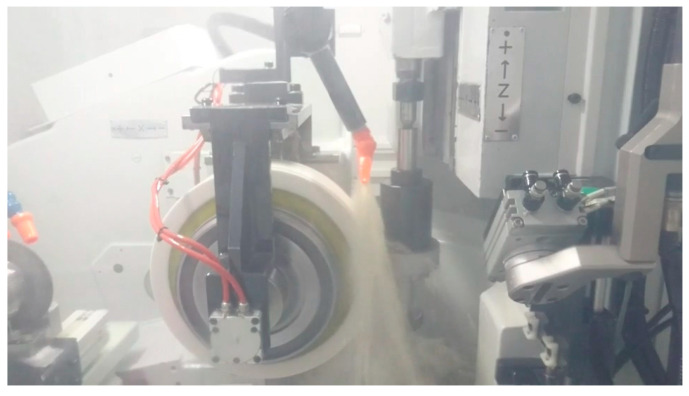
Photograph of grinding process.

In the experimental setup, the continuous generating gear grinding machine with an Al_2_O_3_ grinding wheel was set from Table 1 to grind the SCM415 workpiece (Kinik, New Taipei City, Taiwan) until the grinding wheel loading appears. The experiments were repeated 100 times to take the average value. In the experimental trials, the AE_RMS_ signal of the AE sensor was collected, the grinding wheel loading was calculated by using the proposed image processing technique, and a commercial surface roughness tester (Model: SJ-411, Mitutoyo, Kawasaki, Japan) was used to measure surface roughness R_a_ of the ground workpiece and quantify its machining quality. Then these three data were used and compared to find their correlation and evaluate the grinding wheel loading phenomena by applying a data analysis method [13]. Figure 4 shows the photograph of grinding process by using the continuous generating gear grinding machine.

## 3. Experimental Results

### 3.1. AE Signals

**Figure 5:**
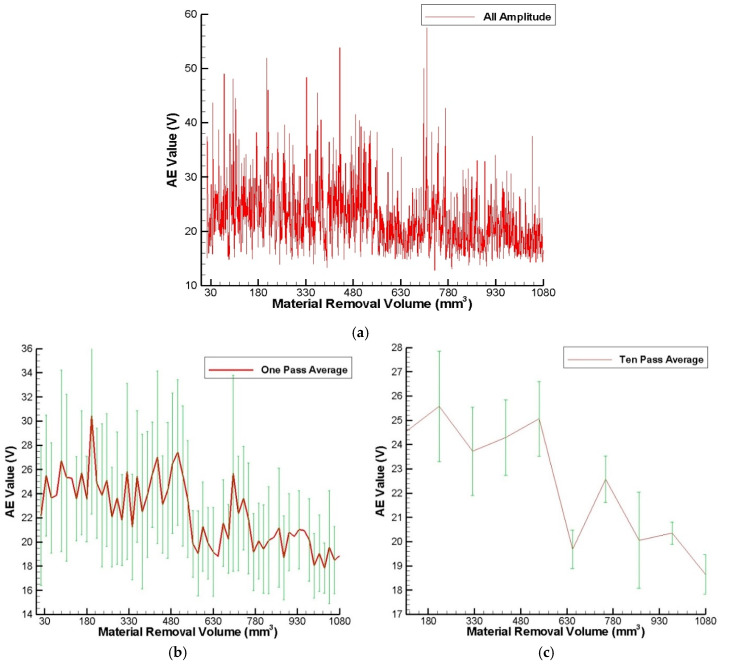
Measured AE signals under 100 repeated experiments (**a**) original data, (**b**) take an average of one experiment, and (**c**) take an average of ten experiments.

Figure 5a–c show the measured AE signals under 100 repeated experiments for taking original data, an average of one experiment, and an average of ten experiments, respectively. It can be seen that the measured original AE signals with a sampling rate of 20 Hz in Figure 5a are too random to observe the trend of the curve. Therefore, averaging is taken to reduce the random errors. As shown in Figure 5b,c it can be observed that both of the curves of the measured AE signals are similar in trend and there are two stages in the grinding process (the first stage and final stage).

In the first stage of grinding, the AE signal increases rapidly and remains stable relatively. After dressing the grinding wheel, the abrasive grains are sharp, so most of the abrasive grains actually grinds the workpiece surface at this stage. In other words, most of the abrasive grains participate in cutting, so the AE signal and grinding force become larger [27].

During the final stage of grinding, the AE signal gradually becomes smaller, because the detached chips more easily accumulate onto the grinding wheel surface, and the grinding ability declines gradually. This phenomenon agrees with the experimental results of the traditional flat surface-grinding machine tool presented in [13]. From the experimental results, the AE signals can be divided into two sub-stages in the final stage of grinding. During the first sub-stage, the accumulated detached chips onto the grinding wheel surface increase and become serious, and the abrasive grains are blunt. This dull process changes the original morphology of the grinding wheel surface, so the grinding ability and AE signal decline significantly. During the second sub-stage, self-sharpening of the grinding wheel happens gradually in the grinding process. The new sharpened abrasive grains of the grinding wheel will improve the grinding ability, so the AE signal and machining efficiency will increase gradually until the loading phenomenon occurs again, and then it returns again to the first sub-stage. Although the first and second sub-stages form a periodic cycle, the overall trend of the AE signal inclines gradually. It means that during the final stage of grinding, the self-sharpening of abrasive grains cannot totally cover the dull abrasive grains. As a result, the grinding wheel must be dressed to remain a good grinding quality and grinding ability before the final stage of grinding.

### 3.2. Proposed Digital Image Processing Technique and Wheel Loading

In order to determine the loading areas over the grinding wheel surface, the captured images of the wheel surface are processed and analyzed by using an improved offline digital image processing technique with an microscope (Model: AM4113T Dino-Lite Premier, AnMo Electronics, New Taipei City, Taiwan ) in this paper. To overcome the metal reflection and segment the metal loading debris from the captured images, HSL (Hue, Saturation, Lightness) color model was used in this paper. HSL and HSV (Hue, Saturation, Value) are alternative representations of the RGB color model, designed in the 1970s by computer graphics researchers to more closely align with the way human vision perceives color-making attributes [44,45]. The procedure of proposed digital image processing technique comprises four basic steps, namely, (1) HSL conversion from RGB color model and binary processing with low L threshold value (0–140), (2) HSL conversion from RGB color model and binary processing with middle L threshold value (140–255), (3) HSL conversion from RGB color model and binary processing with high L threshold value (200–255), (4) overlap processing, and (5) morphology processing.

**Figure 6:**
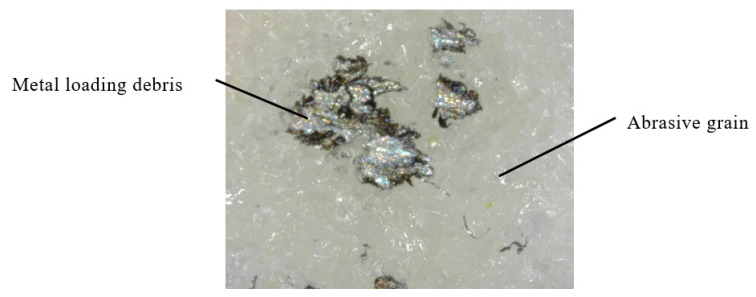
Original captured image of grinding wheel surface.

**Figure 7:**
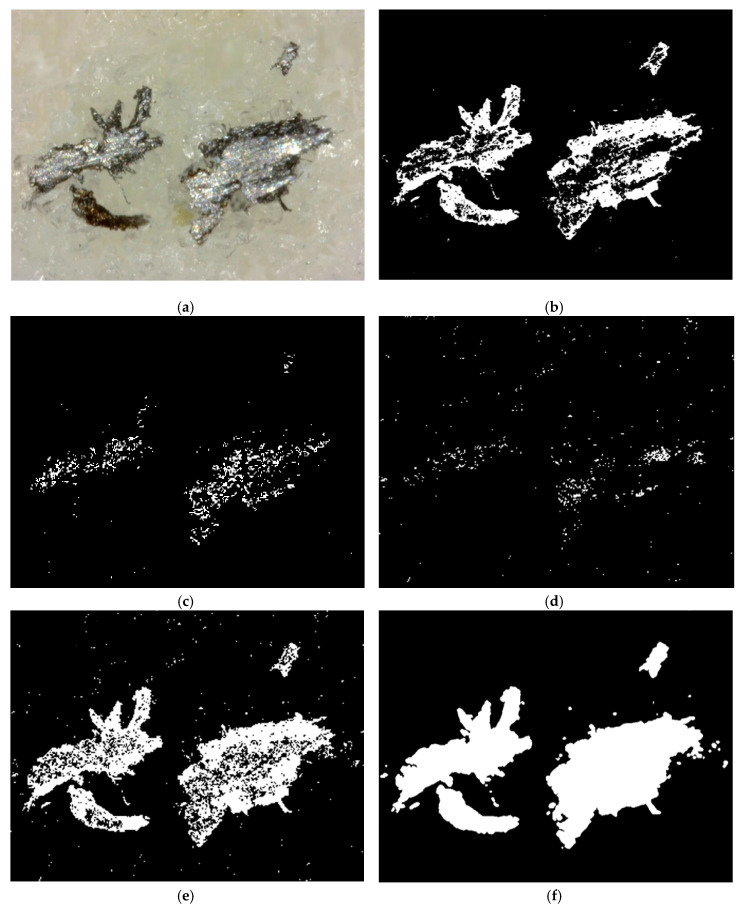
Image processing results: (**a**) original image, (**b**) HSL conversion and binary processing with low L threshold value, (**c**) HSL conversion and binary processing with middle L threshold value, (**d**) HSL conversion and binary processing with high L threshold value, (**e**) overlap processing, and (**f**) morphology processing.

As shown in Figure 6, the metal loading debris is characterized by a wide range of gray levels. Therefore, the metal loading debris cannot be detected by a traditional image processing procedure. In this paper, three different threshold values of L are chosen. Figure 7a shows the original image. Figure 7b–d show the processing results for the HSL conversion and binary processing with low, middle, and high L threshold values, respectively. As shown, some metal loading debris and abrasive grains are misjudged, so a step of overlap processing is used to overcome this problem. For this step, three images in steps (1), (2), and (3) are overlapped into another image, as shown in Figure 7e. Having done so, a simple morphological operation followed by a particle filtering procedure is performed to remove any noise and segment the metal loading debris from the overlapped image, as shown in Figure 7f. It is noted that Ko et al. (2013) and Liu et al. (2013, 2018) provide a more comprehensive description of the morphology processing [13,46,47]. From the comparison of Figure 7a,f it proves that the proposed offline digital image processing technique is workable and it can reliably segment the metal loading debris from the original captured image. 

**Figure 8:**
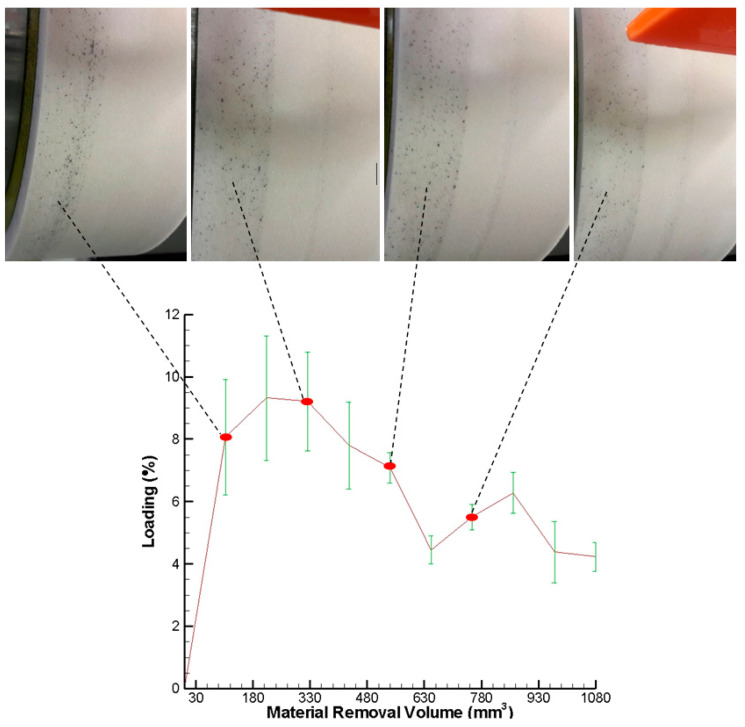
Measured wheel loading over grinding wheel surface and captured wheel loading images.

Consequently, the proposed digital image processing technique and MATLAB image processing functions were used to extract the areas of metal loading debris over the grinding wheel surface and calculate the percentage of the loading amount to the grinding wheel surface, respectively. Figure 8 shows the measured wheel loading over the grinding wheel surface and captured wheel loading images at four different time. As shown, the measured wheel loading and captured wheel loading images match well.

**Figure 9:**
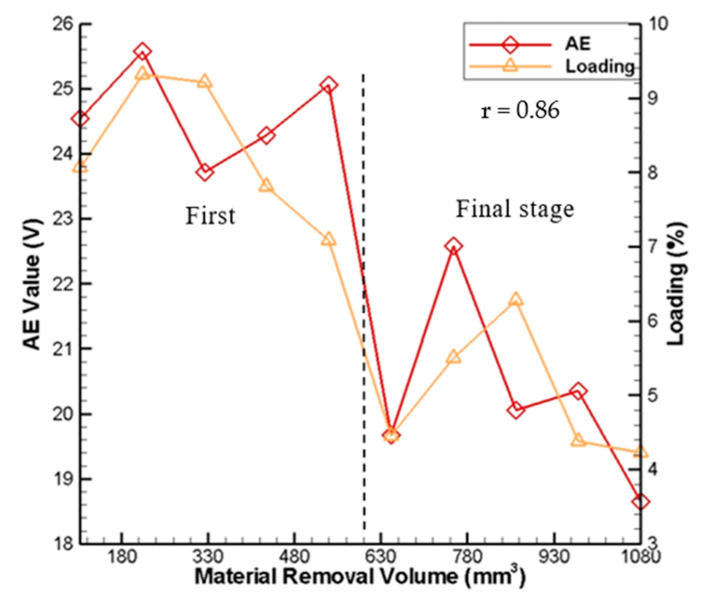
Measured wheel loading over grinding wheel surface and captured wheel loading images.

The comparison of the measured wheel loading and the measured AE signals with the material removal volume is shown in Figure 9. In this figure, the measured wheel loading is sampled ten times, and the average value is taken. As shown, both of the curves of the measured wheel loading and the measured AE signals are similar in trend. Pearson’s linear correlation coefficient r between the measured wheel loading and measured AE signals with the material removal volume is 0.86. During the first stage of grinding, the loading amount over the grinding wheel surface increases rapidly and remains stable relatively. In this stage, when the loading amount is greatest (9.2%), the AE signal is also the largest (25.5 V). During the final stage, just like the AE signals, the loading amount also can be divided into two sub-stages in the final stage of grinding. Although the first and second sub-stages form a periodic cycle, the overall trend of the loading amount decreases gradually to reach a stable variation. As a result, there is a strong positive correlation between the grinding wheel loading amount and the AE signals for the continuous generating gear grinding machine based on the experimental results.

### 3.3. Surface Roughness

**Figure 10:**
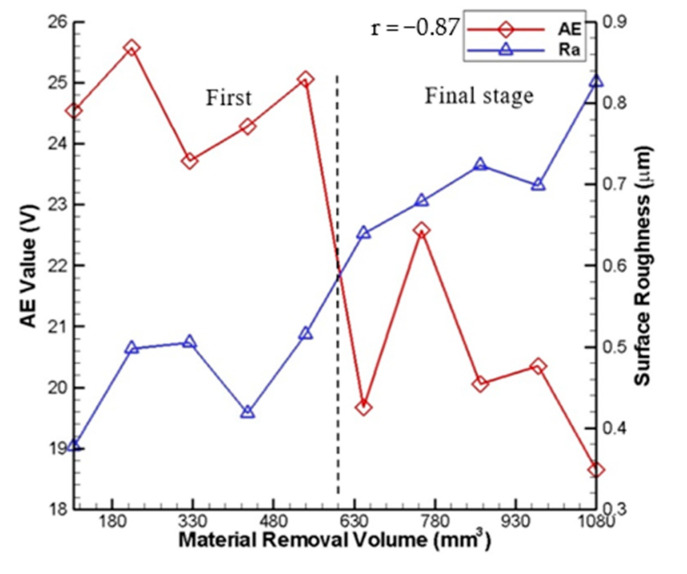
Comparison of measured AE signals and measured surface roughness with material removal volume.

**Figure 11:**
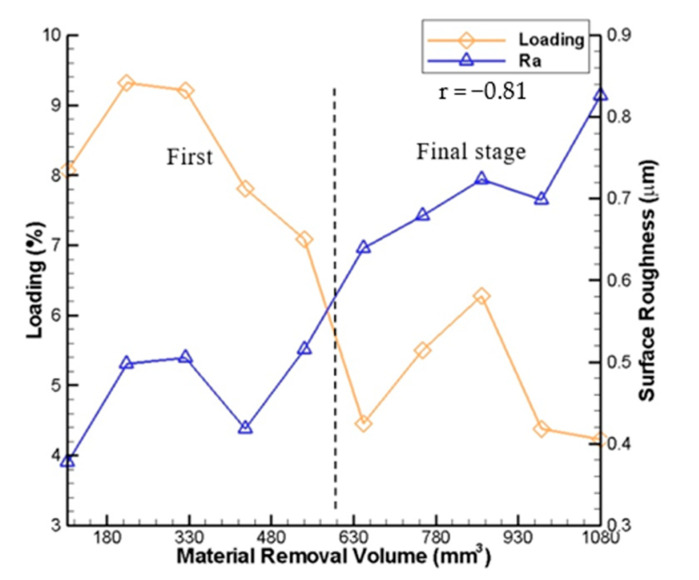
Comparison of measured wheel loading and measured surface roughness with material removal volume.

The comparisons of the measured surface roughness R_a_ as well as the measured AE signals and the measured wheel loading with the material removal volume are shown in Figure 10 and Figure 11, respectively. In these figures, the measured surface roughness R_a_ is also sampled ten times, and the average value is taken. As shown, during the first stage of grinding, the measured surface roughness R_a_ remains a stable variation (between 0.38 and 0.58 μm). During the final stage of grinding, the measured surface roughness R_a_ gradually increases with the material removal volume (between 0.58 and 0.82 μm). It may be attributed to the decline of the grinding ability due to the accumulation of the detached chips onto the grinding wheel surface and the deterioration of grinding quality due to contamination of the grinding wheel. Therefore, the grinding quality of the workpiece in the first stage is better than that in the final stage. It can be observed that the curve of the measured surface roughness R_a_ has an opposite trend when compared to those of the measured wheel loading amount and AE signals. In Figure 10, Pearson’s linear correlation coefficient r between the measured AE signals and measured surface roughness with the material removal volume is −0.87. In Figure 11, Pearson’s linear correlation coefficient r between the measured wheel loading and measured surface roughness with the material removal volume is −0.81.

As a consequence, during the grinding process of the continuous generating gear grinding machine based on the experimental results, two stages of AE signals, the loading amount over the grinding wheel surface, and surface roughness R_a_ are observed and identified, respectively. During the first stage of grinding, the AE signal and the loading amount over the grinding wheel surface increase rapidly and remain stable relatively, and the grinding quality of the workpiece is better. During the final stage of grinding, the overall trend of the AE signal inclines gradually, the grinding quality of the workpiece is the worst, and the loading amount decreases gradually to reach a stable variation. The AE signal of 22 V (at the end of the first stage of grinding, material removal volume of 600 mm^3^) is an early warning quantitative index to identify the condition of the grinding wheel, dress it, and improve the grinding quality in this paper.

## 4. Conclusions

In the manufacturing industry, continuous generating gear grinding machines gain rising importance. It is crucial and necessary to develop a monitoring method for the online evaluation of grinding processes for continuous generating gear grinding machines to improve the grinding accuracy and efficiency. Therefore, in the present paper, a measurement method is proposed to identify the grinding wheel loading phenomenon in the grinding process for continuous generating gear grinding machines based on AE signals. In comparison to existing literature, the experimental results have shown that the proposed measurement method can provide an early warning quantitative grinding wheel loading evaluation to dress the grinding wheel and improve the grinding quality based on the AE signals online at fixed grinding parameters for continuous generating gear grinding machines, which is the main contribution of this paper.

## Figures and Tables

**Table 1:** Grinding parameters of proposed measurement method.

Variable	Corresponding Value
Machine	LGA-2812
Spindle speed	2000 rpm
Workpiece axis speed	Auto adjustment according to the grinding wheel speed
Grinding wheel	KINIK 1A32A120J8V, Al_2_O_3_
Workpiece material	SCM415
Feedrate	150 mm/min
Cutting depth	0.03 mm
Cutting coolant	Yes
